# Pasture-finishing of bison improves animal metabolic health and potential health-promoting compounds in meat

**DOI:** 10.1186/s40104-023-00843-2

**Published:** 2023-04-01

**Authors:** Stephan van Vliet, Amanda D. Blair, Lydia M. Hite, Jennifer Cloward, Robert E. Ward, Carter Kruse, Herman A. van Wietmarchsen, Nick van Eekeren, Scott L. Kronberg, Frederick D. Provenza

**Affiliations:** 1 Center for Human Nutrition Studies, Department of Nutrition, Dietetics, and Food Sciences, College of Agriculture and Applied Sciences, Utah State University, Logan, UT 84322 USA; 2 Department of Wildland Resources, Utah State University, Logan, UT 84332 USA; 3 Department of Animal Science, South Dakota State University, Brookings, SD 57707 USA; 4 Turner Institute of Ecoagriculture, Bozeman, MT 59718 USA; 5 Louis Bolk Institute, Bunnik, 3981 AJ the Netherlands; 6 Northern Great Plains Research Laboratory, USDA-Agricultural Research Service, Mandan, ND 58554 USA

**Keywords:** Bison, Grass-fed, Meat, Nutrition, Omega-3 fats, Pasture-raised, Phytochemicals

## Abstract

**Background:**

With rising concerns regarding the effects of red meat on human and environmental health, a growing number of livestock producers are exploring ways to improve production systems. A promising avenue includes agro-ecological practices such as rotational grazing of locally adapted ruminants. Additionally, growing consumer interest in pasture-finished meat (i.e., grass-fed) has raised questions about its nutritional composition. Thus, the goal of this study was to determine the impact of two common finishing systems in North American bison—pasture-finished or pen-finished on concentrates for 146 d—on metabolomic, lipidomic, and fatty acid profiles of striploins (*M. longissimus lumborum*).

**Results:**

Six hundred and seventy-one (671) out of 1570 profiled compounds (43%) differed between pasture- and pen-finished conditions (*n* = 20 animals per group) (all, *P* < 0.05). Relative to pasture-finished animals, the muscle of pen-finished animals displayed elevated glucose metabolites (~ 1.6-fold), triglycerides (~ 2-fold), markers of oxidative stress (~ 1.5-fold), and proteolysis (~ 1.2-fold). In contrast, pasture-finished animals displayed improved mitochondrial (~ 1.3-fold higher levels of various Krebs cycle metabolites) and carnitine metabolism (~ 3-fold higher levels of long-chain acyl carnitines) (all *P* < 0.05). Pasture-finishing also concentrated higher levels of phenolics (~ 2.3-fold), alpha-tocopherol (~ 5.8-fold), carotene (~ 2.0-fold), and very long-chain fatty acids (~ 1.3-fold) in their meat, while having lower levels of a common advanced lipoxidation (4-hydroxy-nonenal-glutathione; ~ 2-fold) and glycation end-product (N6-carboxymethyllysine; ~ 1.7-fold) (all *P* < 0.05). In contrast, vitamins B_5_, B_6_, and C, gamma/beta-tocopherol, and three phenolics commonly found in alfalfa were ~ 2.5-fold higher in pen-finished animals (all *P* < 0.05); suggesting some concentrate feeding, or grazing plants rich in those compounds, may be beneficial.

**Conclusions:**

Pasture-finishing (i.e., grass-fed) broadly improves bison metabolic health and accumulates additional potential health-promoting compounds in their meat compared to concentrate finishing in confinement (i.e., pen-finished). Our data, however, does not indicate that meat from pen-finished bison is therefore unhealthy. The studied bison meat—irrespective of finishing practice—contained favorable omega 6:3 ratios (< 3.2), and amino acid and vitamin profiles. Our study represents one of the deepest meat profiling studies to date (> 1500 unique compounds), having revealed previously unrecognized differences in animal metabolic health and nutritional composition because of finishing mode. Whether observed nutritional differences have an appreciable effect on human health remains to be determined.

**Graphical abstract:**

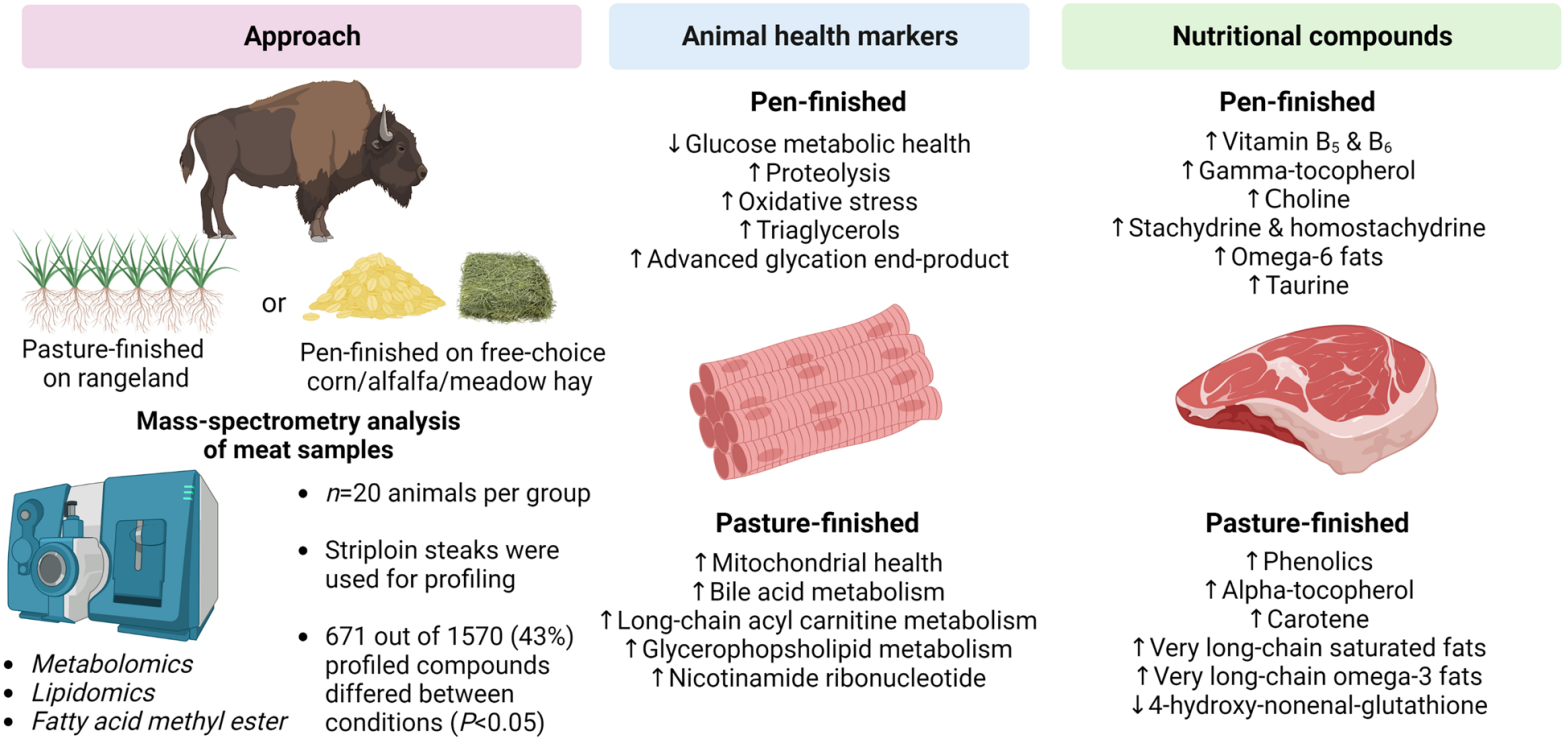

**Supplementary Information:**

The online version contains supplementary material available at 10.1186/s40104-023-00843-2.

## Background

Bison are a quintessential symbol of the North American Great Plains. Once considered a main source of red meat in the pre-industrial American diet [1], bison meat is currently growing in popularity with North American consumers. Retail sales of bison meat have more than doubled in the past decade and reached $350 million in 2017, while continued sale increases are predicted over the coming years [2].

Much like beef, bison meat can be from animals finished on grains in confinement or on pasture (i.e., grass-fed), though bison are typically provided with less grains and more space to roam in confined finishing operations compared to cattle. Previous studies in cattle, goats, and sheep have reported that pasture-finishing can improve animal welfare [3, 4] and transfers phytochemicals—with potential anti-inflammatory and anti-oxidant effects—from forage to animal [5, 6].

As concerns regarding the effects of red meat on human and environmental health continue to mount [7], a growing number of producers are seeking ways to improve environmental and human health by implementing agro-ecological principles (more closely mimicking natural systems in farming) [8]. For example, adaptive grazing of bison on biodiverse rangelands was found to improve soil carbon levels and ecosystem function [9, 10]. Despite potential ecosystem and animal welfare benefits, it is currently unknown if finishing bison using such practices confers benefits to the nutritional compounds in their meat.

Thus, the goal of this work was to provide insight into meat nutritional composition and animal metabolic health by performing multi-omics and fatty acid analysis on bison meat from animals finished on rangeland pasture (pasture-finished group) vs. meat from animals finished in confinement that were fed free-choice whole shelled corn, meadow hay, and alfalfa hay (pen-finished group).

## Methods

### Animal finishing methods and sample collection

Rearing and harvesting of bison was performed in accordance with relevant guidelines and regulations set forth by the United States Department of Agriculture (USDA). Since this work evaluated bison from commercial ranches, and did not involve intervention by the research team, Institutional Animal Care and Use Committee (IAUAC) approval was not necessary. All bison bulls (*Bison bison bison*) grazed native rangelands in the Sandhills of Northern Nebraska (McGinley Ranch) from weaning age prior to the finishing period. Prior to the finishing period, animals were rotationally grazed with 1200 yearling bison and moved every 2 to 5 d amongst pastures, while averaging ∼27 ha with a stocking density of ∼16,812 kg/ha. Typical plant composition of these rangeland pastures is described in Additional file 1.

After grazing rangeland pastures during the weaning and stocker phase (from late 2018 until June 2021), the entire herd was run through a chute and bulls were randomly split as follows: one group went into a pen and was provided ad lib access to meadow hay, alfalfa hay bales, and whole shell corn prior to harvest (pen-finished group), while a second group of bulls were continued to be rotationally grazed on pasture until harvest (pasture-finished group). Both groups did so for 146 d until being slaughtered in November 2021.

During the pen-finishing period, bulls were kept in loose confinement with approximately 275 m^2^ of space per animal. On average, pen-finished bulls consumed 7.3 kg/d of corn, 5.1 kg/d of alfalfa and 2.0 kg/d of meadow hay, which was available as a free-choice arrangement in separate feeding bunks. Pasture-finished bulls had access to a salt block on pasture, but did not consume a vitamin/mineral formulation. Average daily gain of the pen- and pasture-finished bulls was 0.75 kg/d and 0.67 kg/d, respectively. Dry matter intake for the pen- and pasture-finished bulls was estimated by the ranch supervisors to be ∼3.2% of bodyweight and ∼3% of bodyweight, respectively.

All bulls were harvested in USDA-inspected slaughter facilities at approximately 30 months of age. Striploins were removed from one side of each carcass approximately 24 h post-harvest, vacuum packaged, and transported in a refrigerated trailer to South Dakota State University’s Meat Laboratory. An approximately 7.5-cm section of the posterior end of 30 striploins (*M. longissimus lumborum*) from each treatment (pasture-finished vs. pen-finished) were sent overnight from South Dakota State University to the Duke Molecular Physiology Institute (corresponding author’s research home at the time) on dry ice. Samples arrived frozen and were immediately transferred to a −40 °C freezer prior to analysis. Twenty randomly chosen striploin samples from each group (pasture-finished; *n* = 20 vs. pen-finished; *n* = 20) were used for analysis and further processed as described below. In our statistical model, the pasture- and pen-finished groups were considered as experimental units representing the study population, while 20 individual animals per group were considered as the sampling units that make up the two experimental units.

For our power calculations on sample size, we assumed a *P*-value ≤ 0.05, a *Q*-value ≤ 0.3, and a standard deviation of 0.2 based on previous work using untargeted metabolomics profiling of beef samples [11]. An *n* = 20 meat samples per group was expected to provide true discovery rates ranging from 93% to 98% assuming differences in 200–240 metabolites. Table 1 describes the abbreviations and group designations used throughout this manuscript. Members of the research team conducting the metabolomics, lipidomics, and fatty acid analysis were blinded to the experimental groups.

**Table 1:** Group identification

Group ID	Description	***n***
Bi_pasture	Pasture-finished on native rangelands; ground and cooked meat	20
Bi_pen	Pen-finished on meadow hay, alfalfa, and corn; ground and cooked meat	20

### Sample processing

All striploins were thawed and trimmed of excess external fat, connective tissue, and accessory muscles. Striploins were ground individually in a commercial meat grinder (Costway Model# EP24739, Ontario, CA, USA) and patties (112 g) were formed from each individual striploin. The meat grinder was thoroughly cleaned with warm water and soap, and dried using paper towels between grinding of individual steaks to avoid contamination. The remaining meat from each striploin was pooled and stored at −40 °C for proximate analysis using AOAC methods (Microbac Laboratories, Warrendale, PA, USA).

All individual striploin patties were cooked in a commercial oven (175 °C) until the internal temperature of the patties registered at 71 °C as determined by a meat thermometer. Two-gram microcore samples were obtained from the middle of each patty using a bioptome device, immediately frozen in liquid nitrogen, and stored at −80 °C until analysis. Samples were analyzed for untargeted metabolomic and complex lipid profiling through collaborations with Metabolon (Morrisville, NC, USA) and analyzed for fatty acid contents at the Department of Nutrition, Dietetics, and Food Sciences, Utah State University (Logan, UT, USA). These analyses are described below.

### Metabolomics profiling

**Fig. 1:**
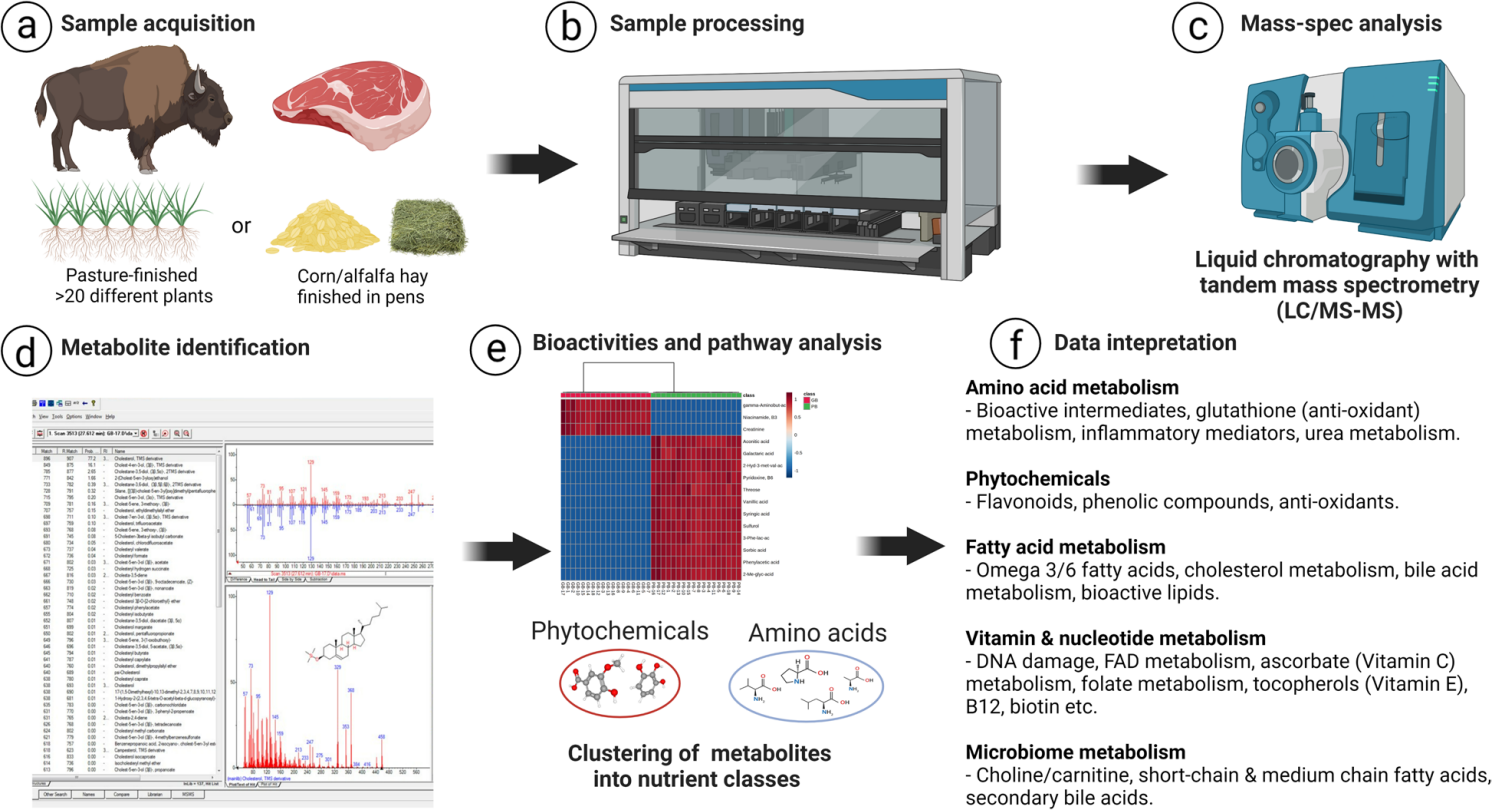
Schematic description of sample preparation and metabolomics analysis. **a** Bison were finished on rangeland pasture (pasture-finished) or fed free choice meadow hay, alfalfa hay, and whole shelled corn (pen-finished). **b** Meat samples (striploins) were cooked and ground, and extractions were performed using the automated MicroLab STAR® system. **c** Subsequent metabolomic analysis was conducted via LC-MS/MS. **d** Metabolites were identified using data extraction and peak identification software, and (**e**) data visualization tools including Metabolon Pathway Explorer, ChemRICH, and Metaboanalyst were used to inform (**f**) data interpretation including potential bioactivities and health effects of identified metabolites

A schematic representation of the study flow is provided in Fig. 1. Sample preparation was carried out as described previously [12]. Briefly, cooked meat samples were weighed and recovery standards were added prior to the first step in the extraction process for quality control purposes. To remove protein and to recover chemically diverse metabolites, proteins were precipitated with methanol under vigorous shaking for 2 min (Glen Mills Geno Grinder 2000, Clifton, NJ, USA) followed by centrifugation (15,000 × *g*). The resulting extract was divided into five fractions: two for analysis by two separate reverse phase (RP)/UPLC-MS/MS methods with positive ion mode electrospray ionization (ESI), one for analysis by RP/UPLC-MS/MS with negative ion mode ESI, one for analysis by HILIC/UPLC-MS/MS with negative ion mode ESI, while one sample was reserved for backup. Sample extracts were placed briefly on a TurboVap® (Zymark, Hopkinton, MA, USA) to remove the organic solvent and subsequently stored overnight under nitrogen before preparation for analysis.

The UPLC-MS/MS platform utilized a Waters Acquity UPLC with Waters UPLC BEH C18-2.1 mm × 100 mm, 1.7 μm columns and a Thermo Scientific Q-Exactive high resolution/accurate mass spectrometer interfaced with a heated electrospray ionization (HESI-II) source and Orbitrap mass analyzer. One aliquot was analyzed using acidic positive ion conditions, which was chromatographically optimized for more hydrophilic compounds. In this method, the extract was gradient eluted from a C18 column (Waters UPLC BEH C18-2.1 mm × 100 mm, 1.7 μm) using water and methanol, containing 0.05% perfluoropentanoic acid (PFPA) and 0.1% formic acid (FA). Another aliquot was also analyzed using acidic positive ion conditions; however, it was chromatographically optimized for more hydrophobic compounds. In this method, the extract was gradient eluted from the same aforementioned C18 column using methanol, acetonitrile, water, 0.05% PFPA and 0.01% FA. Another aliquot was analyzed using basic negative ion optimized conditions using a separate dedicated C18 column. The basic extracts were gradient eluted from the column using methanol and water with 6.5 mmol/L ammonium bicarbonate at pH 8. The fourth aliquot was analyzed via negative ionization following elution from a HILIC column (Waters UPLC BEH Amide 2.1 mm × 150 mm, 1.7 μm) using a gradient consisting of water and acetonitrile with 10 mmol/L ammonium formate at pH 10.8. The MS analysis alternated between MS and data-dependent MS^n^ scans using dynamic exclusion, while the scan range covered *m/z* 70–1000 at a resolving power of R = 35,000 optimized at 50% of the maximum peak height (FWHM).

Metabolites were identified by automated comparison of the ion features in the samples to a reference library of chemical standard entries that considered the retention time, molecular weight (*m/z*), preferred adducts, in-source fragments, and associated MS spectra to properly distinguish metabolites. The data were curated by visual inspection for quality control using Metabolon’s proprietary software. Library matches for each compound were checked for each sample and corrected if necessary. Peaks were quantified using area-under-the-curve. A data normalization step was performed to correct for variation resulting from instrument inter-day tuning differences by setting the medians to equal one (1.00) and normalizing each data point proportionately (termed “block correction”). This preserved variation between samples but allowed metabolites of widely different raw peak areas to be compared on a similar graphical scale.

### Lipidomics profiling

Samples were weighed and then soaked in 1:1 dichloromethane:methanol overnight at 4 °C. The supernatants were subjected to a Bligh-Dyer extraction [13] using methanol/water/dichloromethane in the presence of deuterated internal standards. The extracts were concentrated under nitrogen and reconstituted in 0.25 mL of 10 mmol/L ammonium acetate dichloromethane:methanol (50:50). The extracts were transferred to inserts and placed in vials for infusion-MS analysis, performed on a Shimadzu LC (Canby, OR, USA) and a Sciex SelexIon-5500 QTRAP (Framingham, MA, USA). The samples were analyzed via both positive and negative mode electrospray. The 5500 QTRAP scan was performed in MRM mode with a total of more than 1100 MRMs. Individual lipid species were quantified by taking the peak area ratios of target compounds and their assigned internal standards, and multiplied by the concentration of the internal standard added to the sample. Lipid species concentrations were background-subtracted using the concentrations detected in process blanks (water extracts) and run-day normalized (when applicable). The resulting background-subtracted, run-day normalized lipid species concentrations were then used to calculate the lipid class concentrations.

### Fatty acid profiling

Fatty acid methyl ester (FAME) analysis was carried out as described previously with slight modifications [14]. One hundred mg of wet-weight cooked meat sample was weighed into a screw-cap glass vial along with a 100 μL internal standard solution of tridecanoic acid (0.5 mg/mL in methanol; T-135; Nu-Chek Prep, Inc., Elysian, MN, USA), 70 μL of 10 mol/L KOH in water, and 530 μL of methanol. The vial was subsequently sealed with a polypropylene-lined cap (ThermoFisher Scientific, Waltham, MA, USA) and placed in a shaking water bath (catalog number 67120; Precision Scientific, Chicago, IL, USA) for incubation at 55 °C for 1.5 h. Samples were subsequently cooled on ice and 580 μL of 24 mol/L H_2_SO_4_ was added. Samples were then vortexed at high speed for 1 min and again incubated with shaking at 55 °C for 1.5 h. One milliliter hexane was used to extract FAME before analysis by gas chromatography (GC). Separation of FAME was performed by a Shimadzu GC-2010 equipped with a HP-88 capillary column (100 m × 0.25 mm, 0.20 μm; Agilent Technologies, Palo Alto, CA, USA) and a flame ionization detector (FID). The gas chromatograph was operated based on the conditions described previously [15]. The column head pressure was 195.6 kPa and the total flow rate was 129.1 mL/min (column flow: 2.47 mL/min; purge flow: 3.0 mL/min). One microliter of sample was injected with a split ratio of 50:1. The oven method was as follows: 35 °C held for 2 min, then increased to a temperature of 170 °C at a rate of 4 °C/min, held there for 4 min, followed by an increased temperature of 240 °C at a rate of 3.5 °C/min, and then held for 7 min. Hydrogen was used as the carrier gas. The injector and FID were operated at 250 °C. Fatty acids were identified based on the similarity of retention times with GC reference standards (Nu-Chek Prep, Inc. Elysian, MN, USA). Fatty acid concentrations were calculated as a % relative to initial raw wet sample weight.

### Data analysis

Following normalization to mass, log transformation, and imputation of missing values with the minimum observed value for each compound, non-parametric Welch’s two-sample *t*-test was used to assess differences in compounds comparing the pasture-finished bison samples to pen-finished bison samples (experimental units) using 5% as the cut-off for statistical significance (*P* ≤ 0.05). False-Discovery Rate statistics (*Q*-value) on the metabolomic and lipid profiling data were performed as described [16] to account for multiple comparisons. A *Q*-value (*Q* < 0.10) was considered to indicate high confidence in the *t*-test result for the given metabolite. Next principal component (PCA) and random forest analysis (RF) was performed to visualize data sets and identify the top 30 metabolites that discriminated between groups using “Mean Decrease Accuracy” (MDA) as the metric. Subsequently, spearman rank correlations between log transformed data of the variables with PCA loadings > 0.60 and < −0.60 (first principal component) were calculated for the pen-finished and pasture-finished groups, separately. The correlations with values > 0.60 and < −0.60 were visualized for the pen- and pasture-finished correlations using Cytoscape 3.9.1 [17]. Furthermore, correlations of the pen-finished data were subtracted from the correlations of the pasture-finished data. Of these subtracted correlations, the ones with values > 0.50 and < −0.50 were visualized as a network as well. Node degrees (the number of connected nodes) were calculated and represented in this second network as node sizes. The perfuse force directed layout algorithm was used to generate the network topology [18]. These statistical analyses were performed in ArrayStudio/Jupyter Notebook on log transformed data and/or R (http://cran.r-project.org/). Additional cluster analysis was performed using ChemRICH (https://chemrich.idsl.me/) software via structural similarity and ontology mapping through the use of InChiKeys and SMILES. Bioactivities and potential health effects of annotated metabolites were explored by entering Chemical Abstracts Service (CAS) # of individual metabolites in FooDB (https://foodb.ca/) and/or PubChem (https://pubchem.ncbi.nlm.nih.gov/) databases, in addition to performing PubMed and Google Scholar searches for individual compounds. Several aspects of the figures were made using Biorender (https://biorender.com/).

## Results and discussion

### Proximate analysis

The results of the proximate analysis are shown in Table 2. Previous work comparing the influence of finishing mode on carcass traits of bison found that pasture-finished meat contained 1.3 g less fat per 100 g when compared to pen-finished bison [19]. In the current study, pasture-finished bison were also leaner (1.9 g less fat per 100 g) compared to the pen-finished bison. Leaner meat is typical in pasture-finished domesticated [20] and wild ruminants [21] when compared to animals finished on concentrates, which can result from high-energy/starch contents of diets and reduced grazing mobility [22]. Our results are consistent with those findings.

**Table 2:** Proximate analysis averages

Food chemistry	Pasture-finished bison	Pen-finished bison
Protein, %	23.5	22.9
Fat, %	2.7	4.6
Ash, %	0.98	0.97
Moisture, %	73.2	71.2

### Statistical summary and significantly altered biochemicals

The untargeted metabolomics analysis of bison samples identified a total of 537 compounds with 496 compounds of known structure (named biochemicals) and 42 compounds of unknown structure (unnamed biochemicals) (Additional file 2: Table S1). Of the 537 compounds identified, 278 (52%) were different (*P* ≤ 0.05) between the pasture- and pen-finished bison striploin samples (Table 3).

**Table 3:** Untargeted metabolomics and lipidomics profiling comparisons

	Metabolomics	Lipidomics
Welch’s two-sample *t*-test	Bi_pasture Bi_pen	Bi_pasture Bi_pen
Total biochemicals identified	538	1032
Total metabolites, *P* ≤ 0.05	278 (52%)	393 (38%)
Biochemicals (**↑│**↓)	115│163	42│351
Total metabolites, 0.05 < *P* ≤ 0.10	42 (8%)	93 (9%)
Biochemicals (**↑│**↓)	18│24	9│84

Arrows up indicate higher in pasture-finished, arrows down indicate higher in pen-finished

**Fig. 2:**
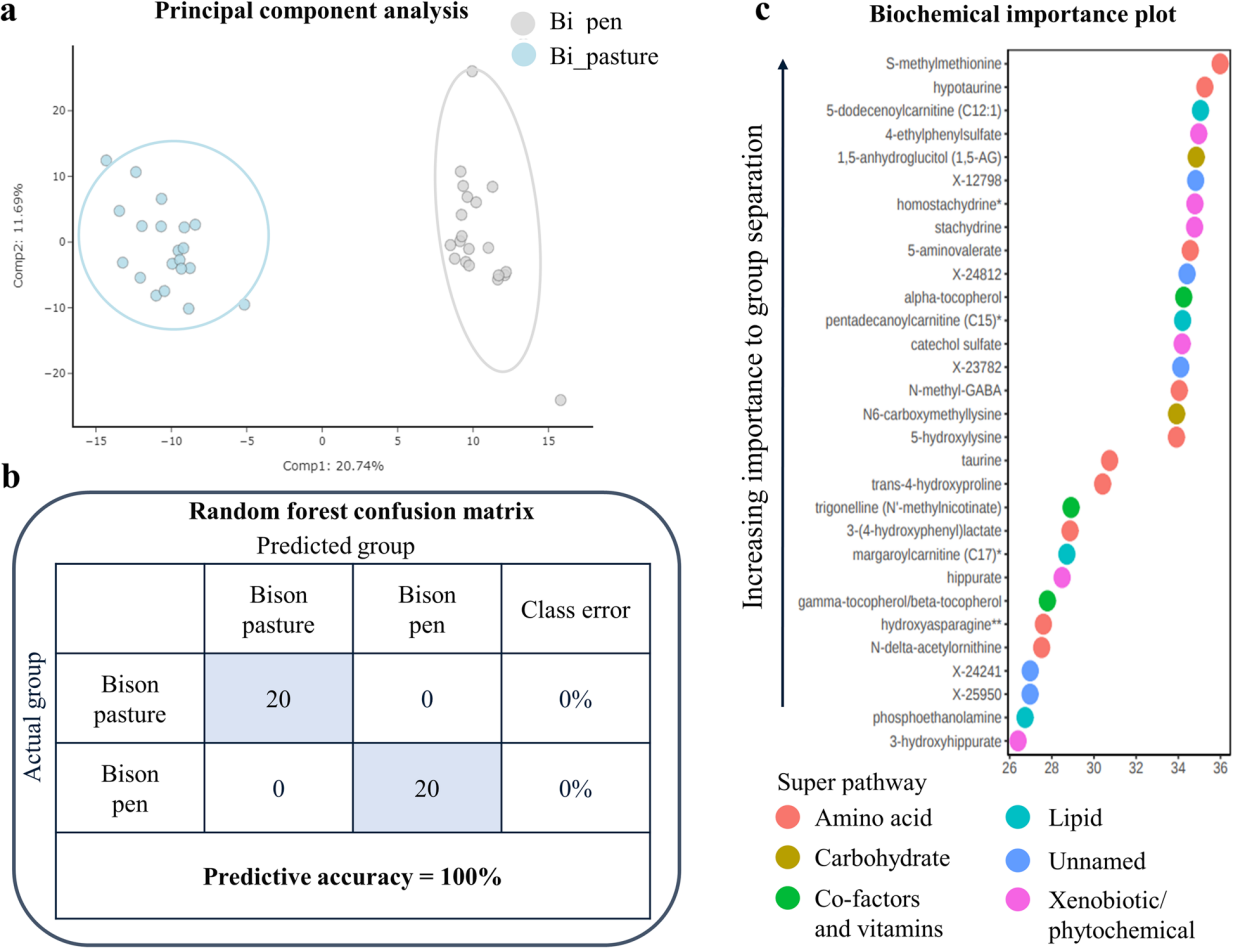
**a** Principal component analysis (PCA) of untargeted metabolite data of pasture-finished (Bi_pasture) and pen-finished (Bi_pen) bison displayed clear and distinct separation between the pasture-finished and the pen-finished samples along component 1 (explaining 20.74% of the variation). **b** Random forest analysis based on the biochemicals detected in this dataset resulted in a predictive accuracy of 100% between dietary conditions. This value is greater than random chance (50%), suggesting these metabolites are of interest as biomarkers. **c** Random forest analyses obtained from grass-fed and grain-fed bison samples. The *y*-axis represents the biochemicals in order of importance for group classification, from top to bottom. The legend represents the type of biochemical and the table next to the chart shows the prediction accuracy based on the random forest results (actual against predicted group)

**Fig. 3:**
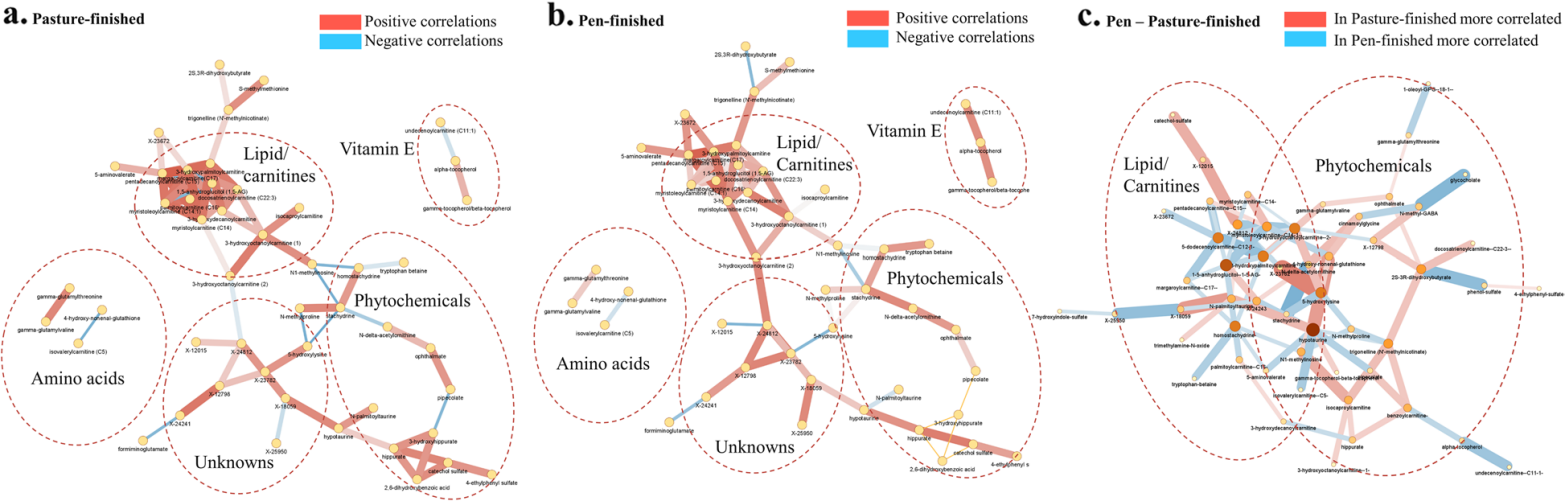
Network visualization of Spearman rank correlations from log transformed data with values > 0.60 and < −0.60 calculated of the variables with PCA loadings > 0.60 and < −0.60 for the pasture-finished (**a**) and pen-finished (**b**) groups separately, and (**c**) pen-finished data subtracted from the correlations of the pasture-finished data. Red color of the edges represents positive correlations with darker red representing higher correlations. Blue represents negative correlations, with darker blue representing higher correlations. Thickness of the edge represents strength of the correlation. The node size represents the degree of the nodes. Groups of related metabolites are marked by the dashed circles

Principal component analysis of the untargeted metabolite data for the bison samples displayed clear and distinct separation between the pasture-finished and the pen-finished samples (explaining 20.74% of the variation along component 1; Fig. 2a). Given that all bison in this study came from the same herd, thus having a similar genetic make-up and diet prior to the 146-d finishing period, we assume that much of the variability captured by this component can be explained by differences in finishing treatments (pasture- vs. pen-finished). In agreement with the separation observed in the PCAs, random forest analysis had a predictive accuracy of 100% (Fig. 2b), further indicating that the biochemical profiles among pasture- and pen-finished bison were distinct from one another with a high degree of confidence. The biochemical importance plot highlights the top 30 metabolites important for the separation between pasture-finished and pen-finished bison (Fig. 2c). The most common metabolite categories represented were amino acids, xenobiotics/phytochemicals, carbohydrates, vitamins, and lipids as indicated by the ChemRICH pathway analysis (Table 4) and network visualizations (Fig. 3). Many of the top metabolites important for the separation of groups included *xenobiotics/phytochemicals* such as hippurate, stachydrine, catechol sulfate, and 4-ethylphenylsulfate; *vitamin metabolites*, including tocopherol (vitamin E precursor) and carotene (vitamin A precursor); *amino acid metabolites* including S-methylmethione and *trans*-4-hydroxyproline, and *lipid metabolites* including long-chain carnitines.

**Table 4:** Differences in metabolite super pathways from bison meat finished on pasture (pasture) or in loose confinement on grain-based concentrates (pen)

Super pathway	Cluster size	*P*-values	FDR	Key compound	Altered metabolites	↑Pasture	↑Pen
Amino acid	162	< 0.001	< 0.001	S-methylmethionine	94	24	56
Lipid	138	< 0.001	< 0.001	5-dodecenoylcarnitine (C12:1)	83	43	33
Carbohydrate	44	< 0.001	< 0.001	Methyl glucopyranoside	29	2	22
Nucleotide	39	< 0.001	< 0.00	N1-methylinosine	24	5	12
Xenobiotics/Phytochemicals	27	< 0.001	0.001	4-Ethylphenylsulfate	16	10	5
Peptide	30	< 0.001	0.001	Phenylacetylglycine	17	8	6
Cofactors/Vitamins	28	< 0.001	0.001	Alpha-tocopherol	13	3	9
Energy	12	0.01	0.01	*L*-Isocitric acid	7	4	1

Analyzed using chemical similarity enrichment analysis software (ChemRICH https://chemrich.idsl.me/home)

The lipidomics profiling analysis of bison samples identified a total of 1032 compounds with 393 (38%) of identified compounds being different (*P* ≤ 0.05) between the pasture- and pen-finished bison striploin samples (Additional file 3: Table S2). Lipid classes responsible for predominant differentiation between samples were triacylglycerols, ceramides, and hexosylceramides. A description of the main metabolite differences within each class and their potential implications is found below.

### Xenobiotic/phytochemical metabolites

**Fig. 4:**
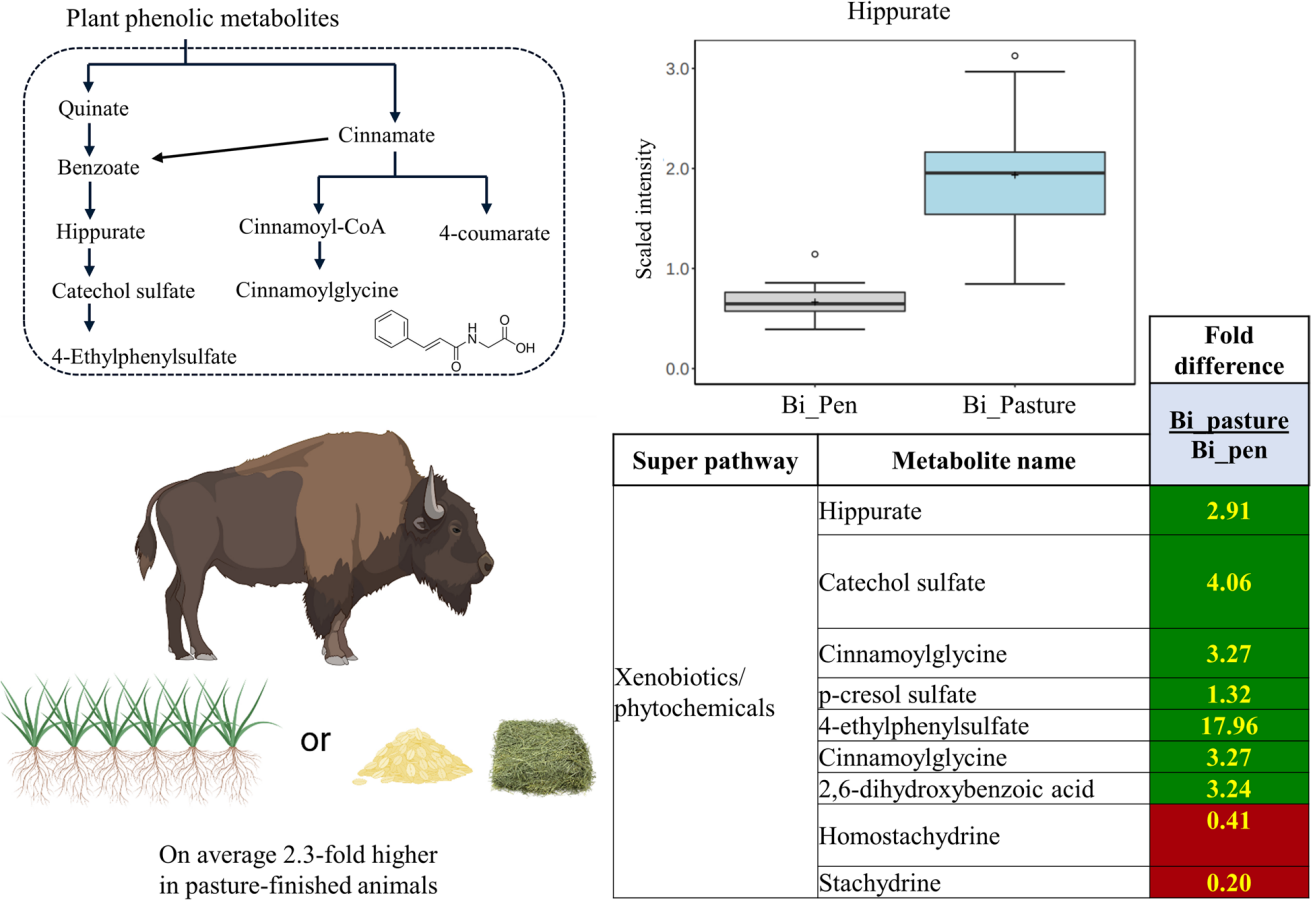
Various plant phenolic compounds, particularly those related to benzoate metabolism, were higher in pasture-finished (Bi_pasture) vs. pen-finished bison (Bi_pen). Homostachydrine and stachydrine, two compounds known to be rich in alfalfa, were higher in pen-finished samples. Dark green colors indicate compounds were higher in pasture-finished bison, while red colors indicate compounds were higher in pen-finished bison. Yellow numbers indicate a significant difference (*P* < 0.05)

Grasses and forbs are rich in antioxidant compounds and vitamins including tocopherols, carotenoids, and other phytochemicals. Phytochemicals are plant-derived secondary compounds that have been studied for their anti-inflammatory and anti-oxidant effects in both livestock [23, 24] and humans [25]. Pasture-finished bison samples showed higher levels of several phytochemical metabolites (Fig. 3), which is in line with previous work performed in cattle, goats, and sheep [5, 6]. Out of the 25 identified phytochemical metabolites, 11 were significantly (*P* < 0.05) elevated in the pasture-finished bison steaks, particularly those related to benzoate metabolism derived from polyphenols in plants (Fig. 4 and Additional file 2: Table S2). Hippurate and its downstream metabolite catechol sulfate were 3- and 4-fold higher, respectively, in the pasture-finished bison. Hippurate is associated with improved gut microbial diversity, metabolic health, and anti-inflammatory status [26]. Cinnamoylglycine, the glycine conjugate of a polyphenol known as cinnamic acid, was 3-fold higher in pasture-finished meat. Cinnamic acid and its metabolites may have anti-inflammatory effects [27, 28] and are linked to a number of health benefits in animal models [29]. 2,6-Dihydroxybenzoic acid, a flavonoid that exhibits anti-oxidant potential in vitro [30], was also found to be 3-fold higher in pasture-finished bison.

While overall phytochemical richness was 2.3-fold higher in pasture-finished animals (Fig. 4 and Additional file 2: Table S1), 5 phytochemical compounds were higher in pen-finished bison. Stachydrine, homostachydrine, and S-methyl cysteine sulfoxide (SMCO)—three compounds concentrated in alfalfa [11, 31]—were 5-, 2.5-, and 1.6-fold higher in the pen-finished bison when compared to the pasture-finished bison, respectively. Animal and in vitro models suggest that stachydrine has anti-oxidant properties and may have brain-protective [32, 33], cardio-protective [34], and anti-cancer effects [35]. Similarly, S-methyl cysteine sulfoxide, a cysteine-containing phytochemical with antidiabetic and antioxidant properties in animal models [36], was also elevated in the pen-finished animals. Two other notable phytochemicals that were higher in pen-finished bison were gluconate and methyl glucopyranoside, which are both glucose derivatives. Their increased presence is likely explained by the higher levels of glucose and glycolytic intermediates in the pen-finished bison (described below). Despite ascribed health benefits of several phytochemicals [25, 37], especially related to their anti-oxidant and anti-inflammatory properties, it is currently unclear if the presence of these compounds in meat impact consumer health as few studies have been conducted comparing products from different grazing systems [38, 39].

### Vitamin metabolites

**Fig. 5:**
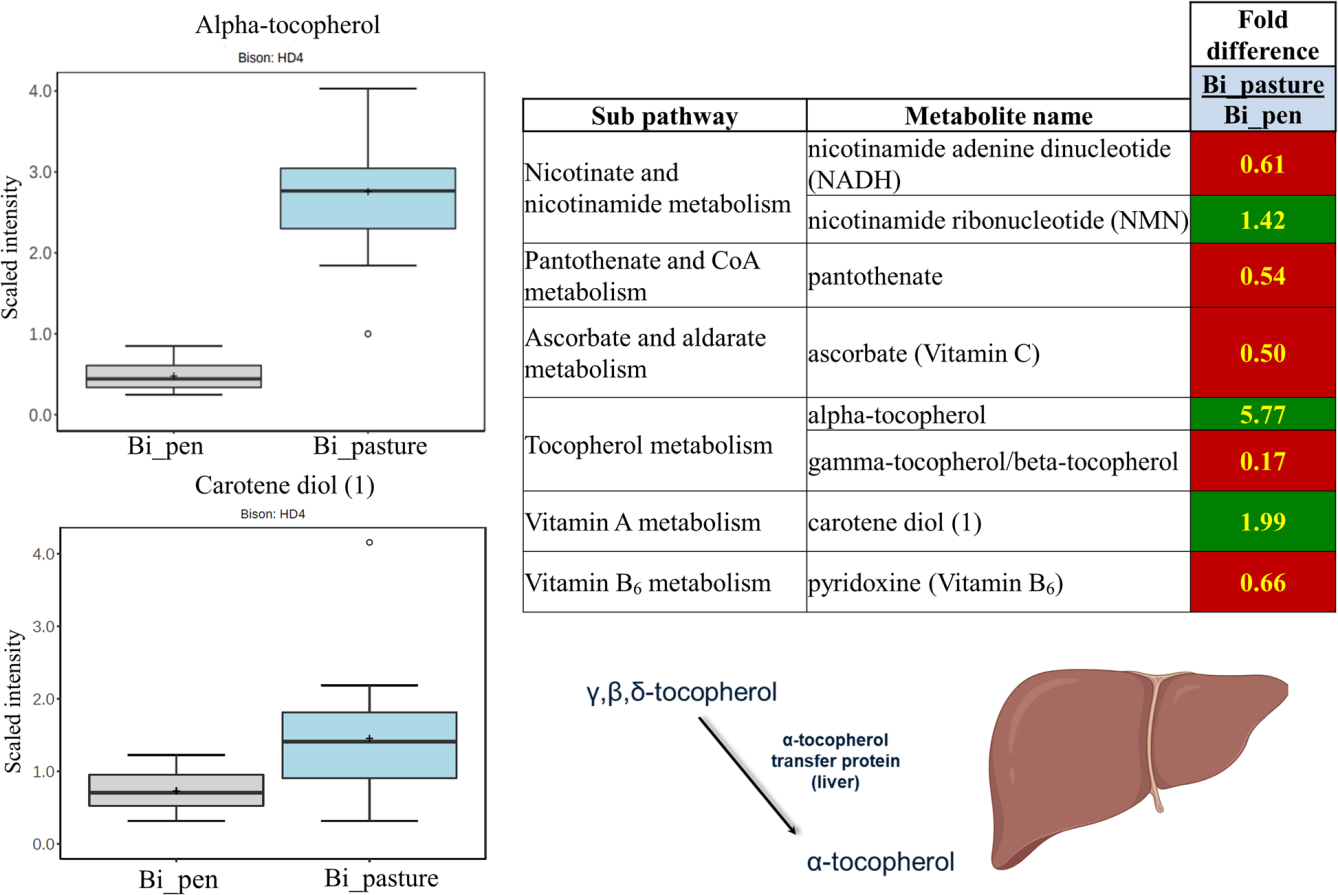
Carotene (vitamin A precursor) and alpha-tocopherol (vitamin E precursor) were higher in pasture-finished bison, while ascorbate (vitamin C), panthotenate (vitamin B_5_), pyrodixine (vitamin B_6_), and gamma/beta-tocopherol were higher in pen-finished bison. Carotene and alpha-tocopherol are highly expressed in forage, while the higher levels of ascorbate, panthotenate, and pyrodixine in pen-finished bison are likely related to its presence in corn, alfalfa, and/or the vitamin/mineral supplement provided as part of the finishing diet. Dark green colors indicate compounds were higher in pasture-finished bison, while red colors indicate compounds were higher in pen-finished bison. Yellow numbers indicate a significant difference (*P* < 0.05)

In line with broader literature on pasture-finished beef and lamb [5], we found that alpha-tocopherol and carotene were 5.8- and 2-fold higher in pasture-finished vs. pen-fed bison meat, respectively (Fig. 5). Carotenoids and tocopherols serve as respective precursors of vitamin A and E [40]. As the effect of grass-feeding on alpha-tocopherol was prominent, these changes likely reflect greater alpha-tocopherol content of the forage and/or increased liver conversion of beta, delta, and/or gamma-tocopherol into alpha-tocopherol by the alpha-tocopherol transfer protein (Fig. 5). The concentrations of gamma/beta-tocopherol were 6-fold higher in the pen-finished bison. This may be the result of the relatively high presence of gamma-/beta-tocopherol in alfalfa [41] and corn [42]. Alpha-tocopherol is the most prevalent tocopherol in mammalian tissue and exhibits higher biological activity and anti-inflammatory properties than gamma- and beta- tocopherol [43]; however, gamma-tocopherol has health benefits that distinguish itself from alpha-tocopherol [44]. We also found that ascorbate (vitamin C) was 2-fold lower in pasture-finished bison. Ruminant muscle meat is generally not considered a good source of vitamin C, and this nutrient is not considered essential for ruminants; however, these findings are noteworthy as higher ascorbate content of meat may improve shelf stability/quality [45].

Nonetheless, we found that putrescine—a common marker indicating reduced freshness of meat [46]—was 1.4-fold elevated in pen-finished samples. As the samples were handled and aged similarly, an explanation for the lower levels of putrescine in the pasture-finished bison may be the increased presence of polyphenols, tocopherols, carotene, which act as antioxidants that can maintain freshness in meat. Alternatively, putrescine is a major amine found in corn and higher levels in the meat from the pen-finished animals may simply reflect a higher dietary intake [47]. Putrescine levels typically remain fairly low in red meat owing to its long shelf life [46], and no organoleptic indication of spoilage of the bison meat was observed by the research team irrespective of the finishing system. While considered a common marker of freshness, the relevance of observed differences in putrescine in our data remains unclear.

Differences in several B-vitamins were also detected. Pantothenate (B_5_) and pyridoxine (B_6_) were almost 2-fold reduced in pasture- compared to pen-finished meat (Fig. 5). This is likely explained by the known abundance of both vitamins in corn. Finally, nicotinamide ribonucleotide (NMN) was significantly elevated in pasture-finished animals. NMN is associated with a host of beneficial health effects in animal studies, including improved glucose and mitochondrial metabolism [48]. As we discuss below, improved glucose and mitochondrial metabolism was found in the pasture-finished animals, which could partly relate to higher NMN levels in the pasture-finished animals.

### Carbohydrate and energy metabolites

**Fig. 6:**
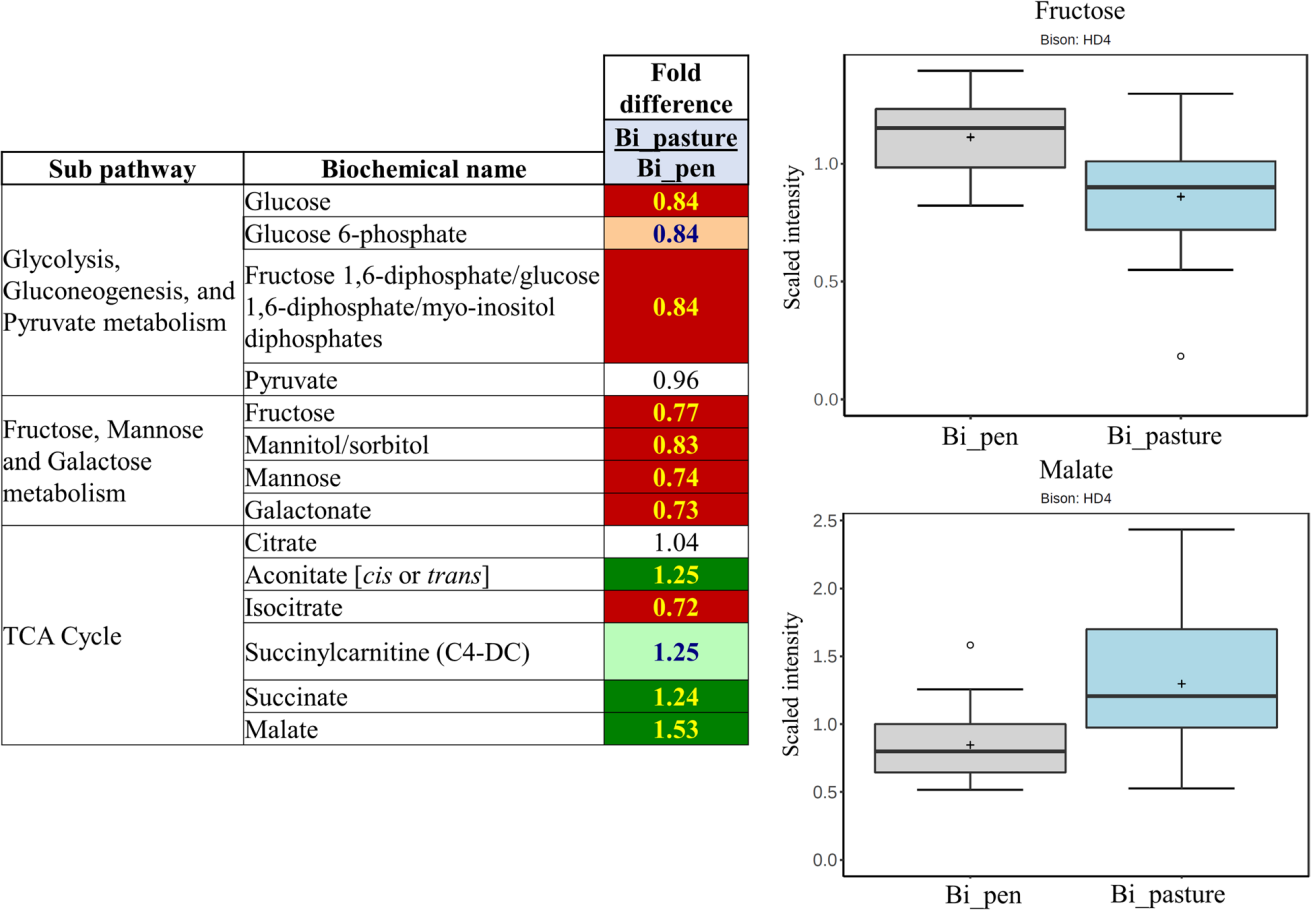
Pasture-finished bison (Bi_pasture) showed improvements in mitochondrial metabolism, while the pen-finished bison (Bi_pen) show potential impairments in glucose metabolism. This likely indicates that pasture-finished bison are metabolically healthier and display more of an athletic phenotype, which is likely the result of their increased ability to engage in physical activity and/or forage feeding. Dark green colors indicate compounds were higher in grass-fed bison, while red colors indicate compounds were higher in pen-finished bison. Yellow numbers indicate a significant difference (*P* < 0.05); blue number and light green/red coloring indicates a trend for differences (*P* < 0.10)

The diet of ruminants significantly impacts energy metabolism, glucose utilization, and glucose availability; these changes are reflected in their muscle tissue. Pasture-finished beef muscle has a higher capacity for oxidative metabolism, as illustrated by increased succinate dehydrogenase activity and elevated Krebs/tricarboxylic acid (TCA) cycle metabolites in prior work [49]. Increased levels of TCA cycle metabolites, including succinate, malate, and citraconate/glutaonate were also identified in the present study (Fig. 6), and higher capacity of oxidative metabolism pasture-finished animals is likely resulting from increased grazing mobility (e.g., ability to roam) and/or decreased concentrate-feeding [22].

Compared to pasture-finished bison, animals finished in pens on concentrates had higher levels of glucose in their muscle. Additionally, fructose, sorbitol, mannonose, and galactonate were higher in the pen-finished animals indicating a more glycolytic muscle phenotype (Fig. 6). A greater reliance on glycolysis is common in faster growing livestock and may relate to higher intake of digestible carbohydrates through concentrate feeding [50]. In humans, increased activity of the sorbitol pathway is considered one of the leading factors in the pathogenesis of glucose intolerance [51]. Impairments in fructose metabolism and elevated glucose levels, similar to our findings, have previously been reported in ruminants with reduced glucose tolerance and impairments in metabolic health [52]. Finally, levels of a common advanced glycation end product (AGE), N6-carboxymethyllysine, were higher in pen-finished samples. The formation of AGEs also plays a role in the pathogenesis of glucose intolerance in humans and other mammals, in part, by stimulating mitochondrial apoptosis and impairing glucose metabolism [53], which is also what we found in the present study.

Certainly not all confined finishing systems are created equal. Previous work in bison reported that offering a choice of feeds (ad libitum choice of whole corn, wheat-middlings, alfalfa hay and/or oat hay) and additional space to roam in loose confinement (334 m^2^/animal) improved average daily gain (ADG) when compared to a more traditional feedlot set-up (21 m^2^/animal) and standardized total mixed rations [54]. In our study, the bison finished in confinement were provided with ad lib access to corn, alfalfa hay, and meadow hay, with a larger than typical space of 275 m^2^/animal. The differences in metabolic health markers between the pen-finished and pasture-finished bison in this study were not as substantial as differences observed between pasture-finished and feedlot-finished cattle using similar metabolomics profiling techniques (to be published observations of the research team), which is likely the result of less grain-feeding and additional space to roam by the pen-finished bison, compared to cattle (which had ∼ 4 times less space). As we studied only two finishing systems, additional work in this area should involve a broader array of finishing options, including comparisons of tight and loose confined feeding, free-choice grain supplemented on pasture, pasture grazing on tame grasses, and pasture grazing on diverse, native forages, as examples.

### Amino acid metabolites

**Fig. 7:**
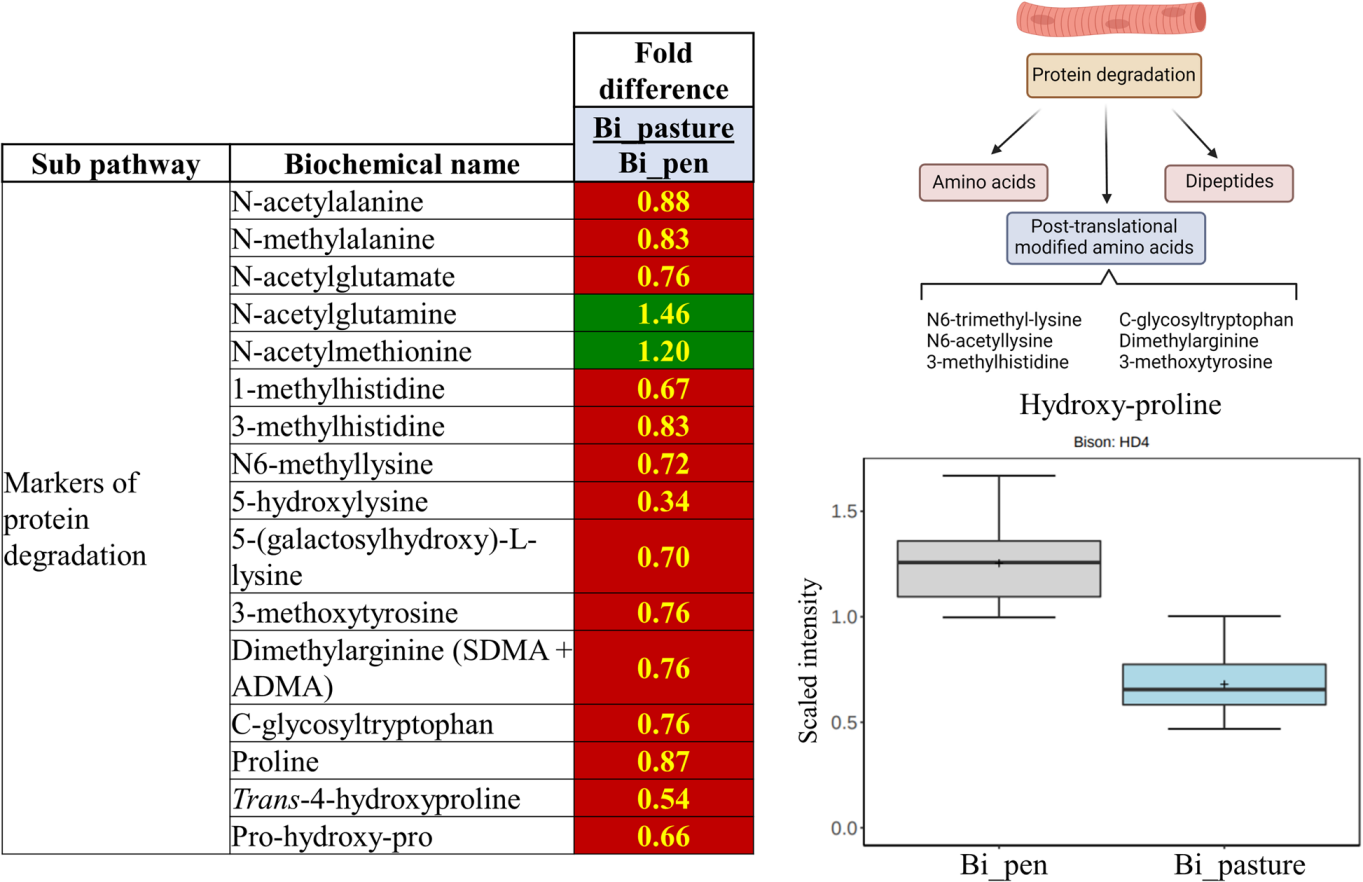
Markers of protein degradation were higher in pen-finished animals. This is likely the result of the reduced ability to engage in physical activity. Dark green colors indicate compounds were higher in pasture-finished bison, while red colors indicate compounds were higher in pen-finished bison. Yellow numbers indicate a significant difference (*P* < 0.05)

Physical activity can impact muscle quality and health. Muscle quality is the result of changes in protein synthesis and breakdown. Several markers of protein breakdown were elevated in the pen-finished bison, including several post-translationally modified N-acetylated amino acids as well as several methylated amino acids, such as 3-methylhistidine, N6,N6,N6-trimethyllysine, and asymmetric dimethylarginine (SDMA) (Fig. 7).

While cooking results in structural changes of protein and post-translational modification, both the pen- and pasture-finished bison patties were cooked for an equal amount of time. Thus, we consider it more likely that the lower levels of proteolysis markers and collagen metabolites in pasture-finished animals are related to metabolism and not cooking. Such metabolites include pro-hydroxy-pro, C-glycosyltryptophan, and trans-4-hydroxyproline (Fig. 7). For example, inactive humans show elevated hydroxyproline levels when compared to active individuals [55]; however, it cannot be ruled out that higher hydroxyproline levels in the muscle of pen-finished animals is also the result of their diet, which may have been higher in hydroxyproline [56]. An alternative explanation for the elevated proteolytic amino acid metabolites could be increased protein turnover in the muscle of pen-finished bison as their average daily gain was greater; however, branched chain amino acids (leucine, isoleucine, and valine) and other essential amino acids were not elevated in the pen-finished samples, which we would expect to be higher in the case of more rapid growth (Additional file 2: Table S1). Our findings are, therefore, in contrast to previous findings in cattle where concentrations of essential amino acids were higher in meat from feedlot as opposed to pastured animals [50].

Noteworthy is that the amino acid taurine was 1.5-fold higher in pen-fished bison. While the impact of finishing mode on taurine has received less attention, previous work found that US Angus cattle finished on grain-based concentrates in confinement contained 1.3-fold less taurine than New Zealand Angus cattle finished on pasture [57]. Our findings are contradictory, as taurine was elevated in the pen-finished bison. The reasons for these conflicting results are currently unknown and future work studying the effects of finishing mode on taurine (and other amino acid such as anserine, carnitine, and creatine with known health benefits that are highly concentrated in meat) in larger data sets is necessary to confirm these findings. No differences were detected in anserine, carnitine, and creatine in this study.

Glutathione levels (both oxidized and reduced) were 1.3-fold higher in pen- vs. pasture-finished animals (Additional file 2: Table S1) (*P* < 0.05). Glutathione is a major intracellular antioxidant in the body of mammals and a higher ratio of oxidized/reduced glutathione is indicative of reduced oxidative stress [58]. Previous work reported that total glutathione levels were higher in grass- vs. grain-fed cattle [59] without impacting the ratio of reduced to oxidized glutathione. This is supported by the present study as the ratio of reduced to oxidized glutathione was not different between pen- vs. pasture-finished bison. The reasons for these differences in glutathione levels are not clear, but previous work suggests that finishing bison on pasture was less stressful (as determined by fecal cortisol concentrations) compared to “loose” or “tight” confinement [60], and our findings appear to support these results.

Further indications of reduced oxidative stress were detected in pasture-finished animals. Uric acid, a potent antioxidant in mammals, was 1.3-fold higher in pasture-finished bison, whereas allantoin was 1.2-fold lower in pasture-finished bison. In the presence of reactive oxygen species, uric acid is metabolized by urate oxidase to allantoin in non-primate mammals [61], and may be considered indicative of increased oxidative stress in ruminants [62]. However, more work is certainly needed to further understand the cause-and-effect relationship of uric acid on mammalian health [63], as elevated uric acid, under pathogenic circumstances, may also indicate increased oxidative stress [64]. Finally, we found 2-fold lower levels of 4-hydroxynonenal-glutathione (4-HNE) in the pasture-finished bison (Additional file 2: Table S1) (*P* < 0.05). A growing body of data implicates 4-HNE is an important effector or biomarker of oxidative stress [65]. This is believed to be partly mediated through mitochondrial dysfunction [66], which is something we also observed in our pen-finished animals.

### Lipid metabolites

Health claims associated with pasture-finished meat often focus on the ratio between omega-6 and omega-3 polyunsaturated fatty acids [5, 20]. Pasture-finished bison had a lower ratio of omega-6 to omega-3 fatty acids (n-6:n-3; 1.8) when compared to pen-finished animals (n-6:n-3; 3.2) (Table 5). With regard to individual omega-3 fatty acids, eicosapentaenoic acid (EPA) and docosapentaenoic acid (DPA) were significantly elevated in pasture-finished bison (Table 5). While we observed statistically significant differences in omega-3 fatty acids between pasture- and pen-finished bison, the omega-6 to omega-3 ratio in pen-finished bison was lower than what is typically reported in studies on grain-fed cattle, which range from 2.8 to 13.6 [20]. The relatively favorable omega-6 to omega-3 ratio in the pen-finished bison is likely because of the free-choice feed arrangement in this study, allowing the animal to self-regulate intake of corn in addition to having access to alfalfa and meadow hay, which are relatively rich in the omega-3 alpha linolenic acid. Ratios in this study are also lower than previous work in bison, which found an omega-6 to omega-3 ratios of 5.7 and 4.6 in grain-fed and pasture-finished bison heifers, respectively [19].

**Table 5:** Fatty acid content of pasture- and pen-finished bison, % of total fatty acids

Carbon #	Fatty Acid	Pasture-finished mean (SD)	Pen-finished mean (SD)
C14:0	Myristic acid	1.33 (0.04)	1.31 (0.06)
C15:0*	Pentadecanoic acid	0.54 (0.02)	0.37 (0.02)
C16:0*	Palmitic acid	17.12 (0.27)	18.42 (0.35)
C16:1n7t*	Palmitoleic acid (*trans*)	0.71 (0.01)	0.36 (0.02)
C16:1n7c*	Palmitoleic acid (*cis*)	2.84 (0.05)	2.32 (0.11)
C17:0	Heptadecanoic acid	1.56 (0.03)	1.53 (0.06)
C18:0*	Stearic acid	19.75 (0.44)	17.87 (0.40)
C18:1 t9	Elaidic acid	0.11 (0.01)	0.58 (0.32)
C18:1n9*	Oleic acid	31.99 (0.60)	38.77 (1.03)
C18:1n7*	Vaccenic acid	1.24 (0.03)	1.51 (0.03)
C18:1n-7 t*	Vaccenic acid (*trans*)	2.34 (0.23)	1.36 (0.09)
CLA, *cis*-9, *trans*-11*	Rumenic acid	0.35 (0.02)	0.16 (0.02)
C18:2n6*	Linoleic acid	4.96 (0.32)	4.81 (0.28)
C18:3n3*	Alpha linolenic acid (ALA)	1.76 (0.09)	0.79 (0.05)
C20:0*	Arachidic acid	0.23 (0.01)	0.12 (0.00)
C20:1n9*	Eicosenoic acid	0.09 (0.00)	0.21 (0.01)
C20:3n6	Dihomo-gamma-linolenic acid	0.23 (0.02)	0.18 (0.01)
C20:4n6*	Arachidonic acid	2.38 (0.17)	1.58 (0.14)
C20:5n3*	Eicosapentaenoic acid (EPA)	0.97 (0.06)	0.53 (0.05)
C22:5n3*	Docosapentaenoic acid (DPA)	1.53 (0.09)	0.76 (0.06)
C24:1n9	Nervonic acid	0.26 (0.03)	0.22 (0.03)
n6-n3 ratio*	–	1.77 (2.13)	3.20 (2.81)

*Indicates a significant difference (*P* < 0.05) between fatty acid concentrations in the pasture- and pen-finished bison

Differences in odd- and longer-chain saturated fatty acids were also observed including pentadecanoate (15:0), stearic acid (18:0), and arachidic acid (20:0), which where elevated in the pasture-finished bison, whereas palmitate (16:0) and oleic acid (18:0) where elevated in the pen-finished bison. Population-based studies [67–69] and randomized controlled trials [70] link higher intakes of palmitate to increased risk of cardiovascular disease relative to other (longer) chain saturated fatty acids such as stearic acid and arachidic acid; with the latter often being associated with a reduced risk of cardiovascular disease [68].

**Fig. 8:**
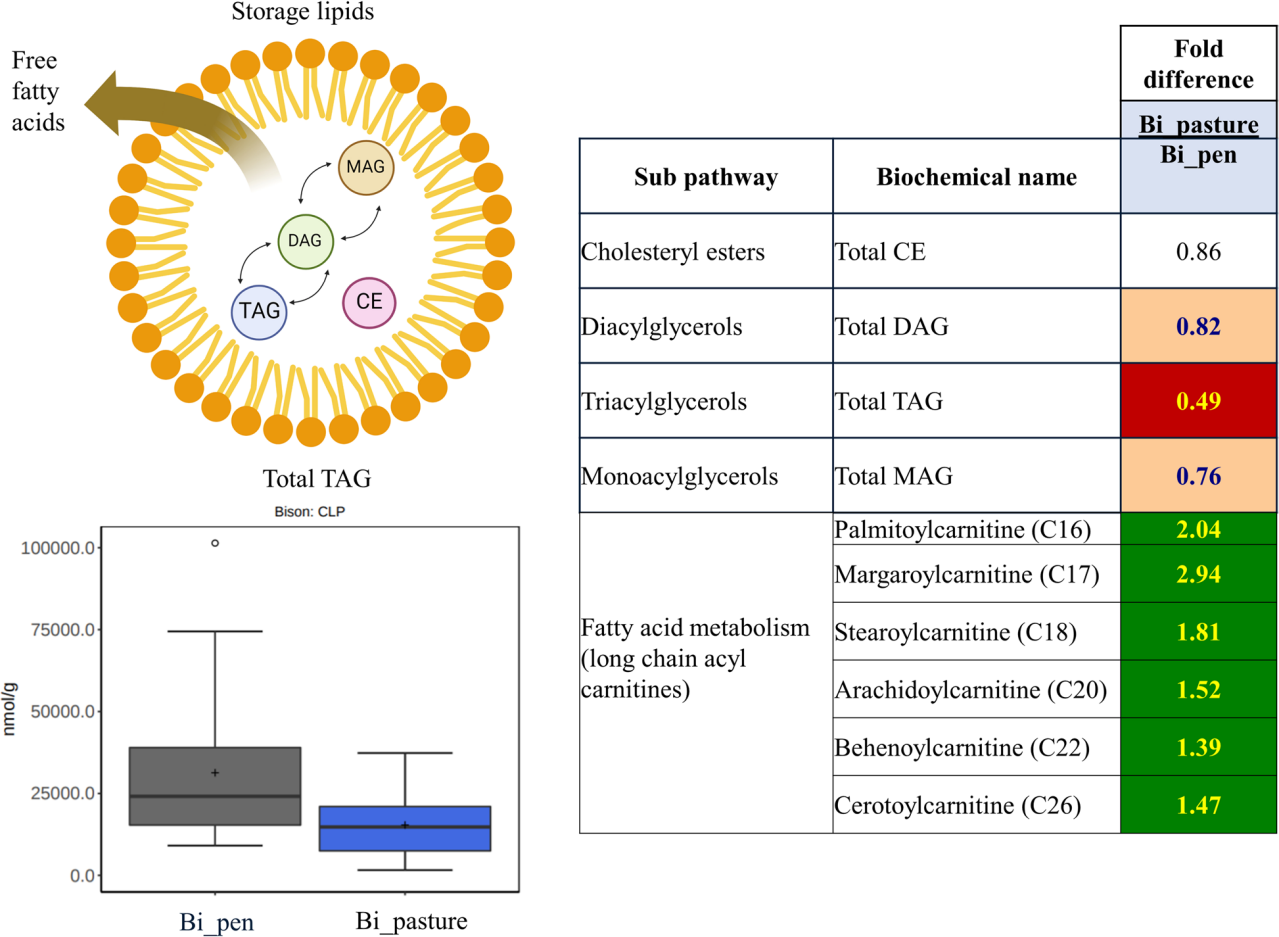
Triacylglycerol (TAG) metabolites were significantly reduced in pasture-finished bison (Bi_pasture) when compared to pen-finished bison (Bi_pen), while long-chain acyl carnitines were elevated. Long-chain acyl carnitines transport fatty acids to the mitochondria for β-oxidation as opposed to storage as lipids. We consider these findings to further reflect that the pasture-finished bison were metabolically healthier as compared to the pen-finished animals. Dark green colors indicate compounds were higher in grass-fed bison, while red colors indicate compounds were higher in pen-finished bison. Yellow numbers indicate a significant difference (*P* < 0.05); blue numbers and light red coloring indicates a trend for differences (*P* < 0.10)

Atherogenic risk can also be related to the fatty acyl glycerol and fatty acyl carnitine content of the meat. Within the bison meat, triacylglycerol (TAG) metabolites were significantly reduced in pasture- compared to pen-finished samples while long-chain acyl carnitines, which transfer long-chain fatty acids across the inner mitochondrial membrane for subsequent β-oxidation, were elevated (Fig. 8). Based on data in non-human mammals [71], we consider these findings to further reflect that the pasture-finished bison were metabolically healthier as compared to the pen-finished animals. No differences in cholesterol metabolites were observed between the pasture- and pen-finished meat.

### Bile acid and glycerophospholipid metabolites

Glycocholate and taurocholate are primary bile acids that aid in the absorption of amino acids, fatty acids, and glucose from the small intestine [72]. Glycocholate and taurocholate were 2.7- and 2.4-fold higher in the muscle of pasture- vs. pen-finished animals. Since bile acids play key homeostatic roles in glucose and lipid metabolism, decreased levels in pen-finished animals may contribute to the negative alterations identified in their glucose metabolism. While these bile acids were measured in the muscle rather than in fecal samples, these results are in agreement with previous findings in cattle where bile acids were measured in fecal material [73]. In that work, similar lower levels of glycocholate and taurocholate where observed in the grain-fed animals relative to pasture-finished animals, with concomitant alterations in glycerophospholipid metabolism [73]. In this work, we found similar upregulations of glycerophospholipid metabolites, including triaglycerols (TAGs), phosphatidylethanolamines (PEs), and sphingolipids such as ceramides (CEs) (Additional file 3: Table S2). In humans and rodent models, higher levels of TAGs, PEs, and CEs are associated with increased cardiovascular risk, glucose intolerance, and oxidative stress [74–76]. Potential causality and the relationship of these in the context of ruminant metabolic health remains to be studied.

## Conclusions

To our knowledge, our work represents the deepest metabolic profiling of bison to date, having identified over 1500 unique compounds. The two primary findings of this study are that pasture-finishing broadly improves metabolic health pathways of bison and increases the presence of potentially health-promoting compounds in their meat. Compared to pasture-finished animals, we observed impairments in glucose metabolism, mitochondrial metabolism, bile acid metabolism, glycerophospholipid metabolism, and increased oxidative stress in pen-finished animals. Several of these metabolic pathways are interrelated and may result from reduced physical activity and/or higher grain-feeding.

However, our data do not per se indicate that bison meat from animal finished in pens is unhealthy to consume. The omega-6:omega-3 fatty acid ratio in the pen-finished bison was favorable when considered in the context of the broader literature on fatty acid profiles in grass-fed vs. grain-fed ruminants [20, 77]. Moreover, some vitamins (B_5_ and B_6_) and phytochemicals (particularly those found in alfalfa) were higher in the pen-finished animals. We, therefore, caution against the simplified narrative of interpretating these data on pasture-finished and pen-finished systems as “good” or “bad”. Red meat, irrespective of whether animals are feedlot- or pasture-finished, can contribute important nutrients to the diet. Moreover, the majority of randomized controlled trials in humans have used grain-fed meat in their interventions (mostly beef), with several studies suggesting moderate intakes may be compatible with good health when overall diet quality is high (sufficiently high in fruits/vegetables/fiber and low in ultra-processed foods) [78]. The importance of overall diet quality should also not be overlooked in discussions on meat from different production systems, as the effects of single foods are rarely binary. However, as compared to pen-finishing on concentrates, pasture-finishing of bison improves markers of animal metabolic health and nutritional compounds in their meat with purported human health benefits. Whether this has an actual appreciable effect on human health remains to be studied in future controlled feeding trials.

There are also additional factors to consider, as pasture-finished meat is more expensive for the consumer than pen/feedlot-finished meat and animals require longer to finish. Nonetheless, agro-ecological approaches, such as the rotational grazing of locally-adapted livestock species (e.g., bison) in this work, are highlighted in the most recent report of the Intergovernmental Panel on Climate Change [79] as potentially promising “nature-based” solutions to address the impact of food production on climate change, in addition to shifting towards more minimally-processed plant foods in the human diet. Future work should, therefore, also attempt to make direct connections between environmental and human health impacts of various production systems, thus linking the health of soils, plants, animals, and humans.

## Supplementary Information

**Additional file 1.** Botanical composition of pastures.

**Additional file 2.** Heat map of metabolites.

**Additional file 3.** Heat map of lipids.

**Additional file 4.** Metabolomics data tables.

**Additional file 5.** Lipidomics data tables.

## Data Availability

The datasets supporting the conclusions of this article are included within the article. In particular, the metabolomics and lipidomics data used for statistical analysis and interpretation can be found in Additional files 4 and 5, respectively. Chromatographic data files can be provided by the corresponding author upon reasonable request.

## Abbreviations

ADMA: Asymmetric dimethylarginine
AGE: Advanced glycation end product
ALA: Alpha linolenic acid
CE: Ceramides
DPA: Docosapentaenoic acid
EPA: Eicosapentaenoic acid
FAME: Fatty acid methyl ester
FID: Flame ionization detector
GC: Gas chromatography
NADH: Nicotinamide adenine dinucleotide
NMN: Nicotinamide ribonucleotide
PCA: Principal component analysis
PE: Phosphatidylethanolamines
RF: Random forest
SMCS: S-methyl cysteine sulfoxide
TAG: Triaglycerol
TCA: Tricarboxylic acid
UPLC-MS/MS: Ultra-high performance liquid chromatography tandem mass-spectrometry
