# Supplementary material for: Pasture-finishing of bison improves animal metabolic health and potential health-promoting compounds in meat
Source: J Anim Sci Biotechnol. 2023 Apr 1;14:49. doi: 10.1186/s40104-023-00843-2 (PMC10067211; doi:10.1186/s40104-023-00843-2)
Supplement: Supplementary file 1 — Additional file 1. Botanical composition of pastures. [file 40104_2023_843_MOESM1_ESM.docx]

**Pasture-finishing of bison improves animal metabolic health and potential health-promoting compounds in meat**

**Stephan van Vliet^1,2^*,** **Amanda D. Blair^3^, Lydia M. Hite^3^, Jennifer Cloward^1^, Robert E. Ward^1^, Carter Kruse^4^, Herman A. van Wietmarchsen^5^, Nick van Eekeren^5^, Scott L. Kronberg^6^, and Frederick D. Provenza^2,6^**

**^1^** **Center for Human Nutrition Studies, Department of Nutrition, Dietetics, and Food Sciences, College of Agriculture and Applied Sciences, Utah State University, Logan, UT, USA 84322**

***Correspondence to:** [**stephan.vanvliet@usu.edu**](mailto:stephan.vanvliet@usu.edu)

*Typical plant composition of rangeland pastures grazed by bison from the weaning phase to finishing on McGinley Ranch in the Sandhills of northern Nebraska.*

Typical warm season grasses included: sand bluestem (*Andropogon hallii*), prairie sandreed (*Calamovilfa longifolia*), switchgrass (*Panicum virgatum*), prairie cordgrass (*Spartina pectinate*), little bluestem (*Schizacrium scoparium*), yellow Indian grass (*Sorgastrum nutans*), blue grama (*Bouteloua gracilis*), buffalo grass (*Buchloe dactylodes*), sand muhly (*Muhlenbergia pungens*), red threeawn (*Aristida comatada*), sand dropseed (*Sporabolus cryptandrous*), sand love grass (*Erigrostis trichodes*), tumblegrass (*Schedonnardus paniculatus*)*,* and lowoutgrass (*Redfeldia flexuosa*).

Typical cool season plants included: needle and thread *(Stipa comata),* western porcupine grass *(Stipa spartea),* green needle grass *(Stipa viridula),* june grass *(Koleria macrantha*), western wheatgrass *(Agropyron smithii*)*,* threadleaf sedge *(Carex filifolia),* sun sedge *(Carex inops), cheatgrass (Bromus tectorum)*.

Typical forbs included: annual sunflower (*Helianthus petiolaris***)***,* stiff sunflower (*Helianthus rigidus*)*,* prickly poppy (*Argemone albiflora*), bush morning glory (*Ipomoea leptophylla*), sweet clover (*Melilotus officinalis),* sand milkweed (*Asclepias arenaria*), american vetch (*Vicia americana*), milkvetch’s (*Astragalous* spp.), locoweeds (*Oxytropis* spp.)*,* silver scurfpea *(Psoralea argophylla*)*,* beardtongue (*Penstemon spp*.), purple prairie clover (*Dalea purpurea)*, dotted blazing star (*Liatris punctata*), wavyleaf thistle (*Circium undulatum*), western ragweed (*Ambrosia psilostachya)*, spiderwort (*Tradescntia occidentalis*), prairie coneflower (*Ratibita columnifera),* rocky mountain bee plant (*Cleome Serrulata*), sand verbena (*Abronia fragrans),* and blue vervane (*Verbena hastata*)*.*

Typical shrubs included: yucca (*Yucca glauca)*, sand cherry (*Prunus pumila*), chockcherry (*Prunus virginiana*), snowberry (*Symphorocarpus occidentalis*), wild rose (*Rosa arkansensis)*, sandbar willow (*Salix exigua*), leadplant (*Amorpha canescens*), prickly pear (*Opuntia polycantha*), Pincusion (*Escobaria missouriensis),* and thimble (*Opuntia fragilis*).
